# Digitally Guided Modified Intentional Replantation for a Tooth with Hopeless Periodontal Prognosis: A Case Report

**DOI:** 10.3390/diagnostics15233080

**Published:** 2025-12-03

**Authors:** Raul Cuesta Román, Ángel Arturo López-González, Joan Obrador de Hevia, Sebastiana Arroyo Bote, Hernán Paublini Oliveira, Pere Riutord-Sbert

**Affiliations:** ADEMA University School, University of the Balearic Islands, 07010 Palma, Spains.arroyo@eua.edu.es (S.A.B.);

**Keywords:** modified intentional replantation, guided tissue regeneration, periodontal regeneration, digital dentistry, tooth autotransplantation, case report

## Abstract

**Background and Clinical Significance:** Advanced periodontitis with severe vertical bone loss and grade III mobility is usually managed by extraction and implant placement. Digital workflows and modern regenerative techniques have opened the possibility of preserving teeth that would traditionally be considered for extraction. This report describes a digitally guided modified intentional replantation (MIR) protocol applied to a maxillary tooth with severe periodontal involvement and unfavourable prognosis. **Case Presentation:** A 68-year-old male, non-smoker, with a history of heart transplantation under stable medical control, presented with generalized Stage IV, Grade C periodontitis. Tooth 21 showed >75% vertical bone loss, probing depths ≥ 9 mm, bleeding on probing, and grade III mobility. After non-surgical therapy and periodontal stabilization, a CAD/CAM-assisted MIR procedure was planned. Cone-beam computed tomography (CBCT) and a 3D-printed tooth replica were used to design a surgical guide for a new recipient socket. The tooth was atraumatically extracted, stored in chilled sterile saline, and managed extraorally for approximately 10 min. Apicoectomy and retrograde sealing with Biodentine^®^ were performed, followed by immediate replantation into the digitally prepared socket, semi-rigid splinting, and guided tissue regeneration using autologous bone chips, xenograft (Bio-Oss^®^), enamel matrix derivative (Emdogain^®^), and a collagen membrane (Bio-Gide^®^). A conventional orthograde root canal treatment was completed within the first month. At 12 months, tooth 21 exhibited grade 0 mobility, probing depths of 3–4 mm without bleeding on probing, and stable soft tissues. Standardized periapical radiographs and CBCT showed radiographic bone fill within the previous defect and a continuous periodontal ligament-like space, with no signs of ankylosis or root resorption. The tooth was fully functional and asymptomatic. **Conclusions:** In this medically complex patient, digitally guided MIR allowed preservation of a tooth with severe periodontal involvement and poor prognosis, achieving favourable short-term clinical and radiographic outcomes. While long-term data and larger series are needed, MIR may be considered a tooth-preserving option in carefully selected cases as an alternative to immediate extraction and implant placement.

## 1. Introduction

Periodontitis is a major cause of tooth loss in adults and has a substantial impact on mastication, aesthetics, and quality of life [1]. Severe forms of periodontitis affect around 10–15% of adults worldwide and are associated with established risk factors such as tobacco use, diabetes, and inadequate plaque control [2,3]. In advanced stages, teeth may present with deep periodontal pockets, extensive vertical bone loss, and increased mobility [4,5], and are often classified with an unfavourable or poor prognosis according to the 2017 AAP/EFP staging and grading system.

Conventional management of teeth with severe bone loss and grade III mobility frequently involves extraction followed by implant-supported rehabilitation [6,7]. However, tooth preservation remains a central goal in periodontology whenever feasible, as it maintains proprioception, avoids the biological and technical risks of implant placement, and can be advantageous in medically compromised patients [8].

Periodontal regeneration aims to restore a functional attachment apparatus by re-establishing new cementum, periodontal ligament (PDL), and alveolar bone [9,10]. Guided tissue regeneration (GTR) and enamel matrix derivative (EMD) have shown favourable clinical and histologic outcomes in intrabony defects and furcations [11,12,13,14]. Nonetheless, when residual bone is severely reduced and tooth mobility is high, conventional regenerative approaches may be limited by mechanical instability and compromised defect morphology [15,16,17].

Intentional replantation (IR) is a technique in which a tooth is deliberately extracted, treated extraorally, and replanted [18,19]. Historically considered a last resort, IR has been revisited with the advent of microsurgical instruments, modern root-end filling materials, and improved regenerative protocols [20]. A modified intentional replantation (MIR) approach combines principles of autotransplantation and GTR, preparing a new recipient socket with improved geometry and stability to enhance clinical outcomes [21,22,23].

Digital dentistry has further refined these procedures. CBCT-based planning, CAD/CAM-fabricated surgical guides, and 3D-printed tooth replicas enable controlled socket preparation and can shorten extraoral time, which is critical for PDL cell viability [24,25,26,27,28]. These protocols may be particularly useful in periodontally compromised teeth, where precise socket design and simultaneous regenerative procedures are required [29].

Evidence on digitally guided MIR in advanced periodontal cases remains scarce, with most publications focusing on autotransplantation of immature teeth or endodontic indications rather than generalized severe periodontitis. There is therefore a need for detailed clinical reports describing protocols and short-term outcomes in such scenarios [30,31,32,33,34,35,36,37,38,39,40,41].

The aim of this case report is to describe a digitally guided MIR protocol applied to a maxillary tooth in a patient with generalized Stage IV, Grade C periodontitis and site-specific severe bone loss and mobility, in the context of heart transplantation and long-term immunosuppression. The report emphasizes clinical decision-making, digital planning, surgical steps, and 12-month clinical and radiographic outcomes.

## 2. Materials and Methods

### 2.1. Study Design and Ethical Aspects

This is a single-patient case report. The procedure was carried out in accordance with the Declaration of Helsinki (2013 revision) and institutional policy for individual clinical cases. According to this policy, formal ethics committee approval is not required for anonymized single-case reports when written informed consent is obtained. The patient provided written informed consent for both the treatment and the publication of anonymized clinical and radiographic data.

### 2.2. Patient Information and Periodontal Diagnosis

A 68-year-old male (born 22 October 1956), non-smoker, with a history of heart transplantation in 2010, was referred for evaluation of a mobile maxillary anterior tooth. The patient was under stable cardiology follow-up and receiving vesimni (6 mg/0.4 mg), balzak plis (40 mg/10 mg/25 mg), lixiana (60 mg), atorvastatin (20 mg), and lovibon (5 mg).

Comprehensive periodontal examination revealed generalized clinical attachment loss and radiographic bone loss extending beyond the middle third of the root at multiple sites, compatible with generalized Stage IV, Grade C periodontitis. Tooth 21 showed probing depths of 7–9 mm with bleeding on probing, >75% vertical bone loss, and grade III mobility (horizontal displacement > 1 mm and detectable vertical mobility). No root fractures, extensive caries, or previous endodontic failures were observed. Residual palatal apical bone and a clinically acceptable crown-to-root ratio were present.

The prognosis of tooth 21 was considered poor/unfavourable based on Kwok and Caton and EFP criteria, taking into account the extent of bone loss, deep pockets, mobility, and the general periodontal condition. However, biologically favourable factors (intact root, residual palatal bone, acceptable crown-to-root ratio, no major endodontic compromise) were also present and contributed to the decision to attempt tooth preservation with MIR rather than immediate extraction.

### 2.3. Initial Periodontal Therapy

Initial therapy consisted of oral hygiene instruction, supragingival and subgingival instrumentation, and occlusal adjustment to reduce traumatic contacts on tooth 21. After periodontal stabilization and improved plaque control, tooth 21 remained with deep pockets and mobility, but inflammation was reduced. These findings supported the consideration of MIR as a tooth-preserving approach.

### 2.4. Digital Planning and Surgical Guide Fabrication

CBCT of the maxilla was obtained to assess defect morphology, residual bone, and anatomical relationships. A digital model of the tooth and surrounding bone was generated by combining CBCT data with intraoral scans. Using CAD/CAM software Version 5.0 (Blue Sky Plan^®^, Blue Sky Bio, Grayslake, IL, USA), a new recipient socket was virtually planned to optimize three-dimensional positioning and primary stability, mimicking an implant-like site adapted to the root morphology.

A 3D-printed replica of tooth 21 (STL model) was fabricated to allow preoperative verification of the planned socket and to reduce extraoral time by rehearsing the fit before replantation. A tooth-supported surgical guide was designed and printed to direct the osteotomy for the new recipient socket.

### 2.5. Surgical Procedure

All procedures were performed under local anaesthesia (4% articaine with 1:100,000 epinephrine). The surgical sequence included:

1. Atraumatic extraction: Tooth 21 was carefully luxated using periotomes to preserve the PDL and surrounding bone. Immediately after extraction, the tooth was stored in chilled sterile saline (approximately 4–6 °C). The total extraoral period was approximately 10 min.
2. Preparation of the recipient socket: After curettage of granulation tissue from the original socket, the CAD/CAM surgical guide was positioned. A guided osteotomy was performed using the implant-type drills from the C3D Cambados guided surgery kit, following the virtual plan. A biologic drilling protocol without irrigation was used to preserve autologous bone chips in the osteotomy walls. These chips were collected and later used as graft material. Drill sequence, diameters, and depth calibration followed the manufacturer’s recommendations and the digital plan, maintaining a stable trajectory and preserving palatal bone for primary stability.
3. Verification with the 3D-printed replica: The printed tooth replica was inserted into the prepared socket to confirm angulation and depth prior to replantation of the original tooth.
4. Extraoral root-end management: During the extraoral phase, apicoectomy was performed, and a retrograde cavity was prepared and sealed with a bioceramic material (Biodentine^®^, Septodont, Saint-Maur-des-Fossés, France) to provide an apical barrier and reduce contamination risk.
5. Replantation and splinting: The original tooth was replanted into the digitally prepared socket, achieving primary stability by adapting to the prepared walls and remaining palatal bone. A semi-rigid splint was placed using fibre-reinforced composite bonded to adjacent teeth, planned for a 4-month period.
6. Guided tissue regeneration: A full-thickness flap was elevated to expose the defect morphology. Autologous bone chips collected during drilling were combined with xenograft particles (Bio-Oss^®^, Geistlich Pharma, Wolhusen, Switzerland) and enamel matrix derivative (Emdogain^®^, Straumann, Basel, Switzerland) and placed around the replanted root. A resorbable collagen membrane (Bio-Gide^®^, Geistlich Pharma, Wolhusen, Switzerland) was positioned to stabilize the grafted area and support space maintenance. The flap was repositioned and sutured to achieve primary closure.

Due to limited intraoperative documentation, only one clinical photograph of the flap design and incision lines was of sufficient quality to be included as an illustrative figure. The limited photographic record is acknowledged as a limitation of the report (Figure 1, Figure 2, Figure 3, Figure 4, Figure 5, Figure 6, Figure 7, Figure 8, Figure 9 and Figure 10).

### 2.6. Postoperative Management and Endodontic Treatment

Postoperative medication included amoxicillin 500 mg every 8 h for 7 days and 0.12% chlorhexidine rinses twice daily for 14 days. The patient was instructed to maintain a soft diet and avoid trauma in the anterior maxillary region.

Once initial soft tissue healing and tooth stability were confirmed, a conventional orthograde root canal treatment was performed within the first postoperative month. The retrograde Biodentine^®^ seal placed during the extraoral phase was not intended to replace full canal disinfection, but to provide an immediate apical barrier until orthograde treatment could be safely completed. This two-step approach (retrograde apical seal during MIR + delayed orthograde obturation) is adopted specifically to prevent internal and inflammatory root resorption, which is a known risk in replanted teeth with untreated canals.

### 2.7. Follow-Up and Outcome Measures

Clinical and radiographic follow-ups were scheduled during 24 weeks and at 12 months postoperatively. Standardized periapical radiographs were taken using fixed film holders to reproduce the projection geometry at each time point. CBCT slices were acquired at baseline and 12 months to assess defect morphology and bone fill.

Primary clinical outcomes included:

- Change in tooth mobility (grade III to grade 0);
- Reduction in probing pocket depth (PPD) to ≤4 mm;
- Absence of bleeding on probing or suppuration.

**Figure 11:**
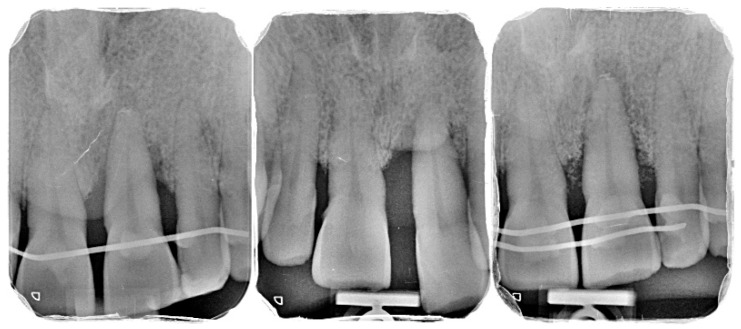
Standardized periapical radiograph, baseline and surgical day.

**Figure 12:**
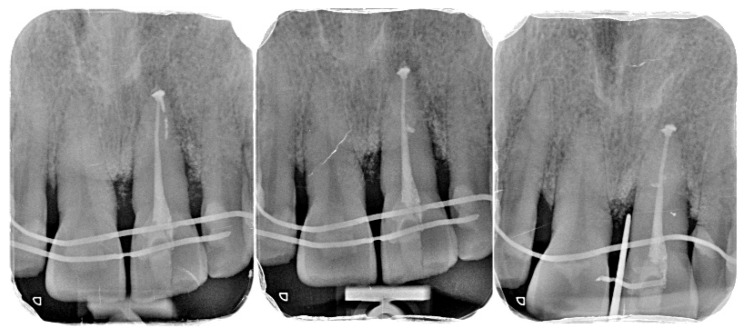
Standardized periapical radiograph, endodontic day and 24 weeks later.

Radiographic outcomes included:

- Radiographic bone fill within the original defect;
- Presence of a periodontal ligament–like space around the root;
- Absence of radiographic signs of ankylosis or root resorption.
- Advanced bone fill with a homogeneous trabecular pattern and stable crestal level; no signs of root resorption or ankylosis. A scale bar is included (Figure 11 and Figure 12).

**Figure 13:**
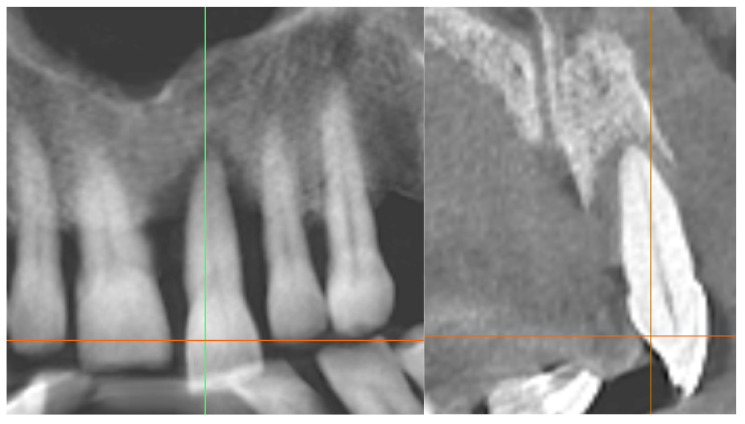
Final clinical outcome at 12 months.

Intraoral view showing periodontal stability, healthy soft-tissue contour, and full functional reintegration of the replanted tooth with balanced occlusion (B/Li orientation indicated) (Figure 13).

**Figure 14:**
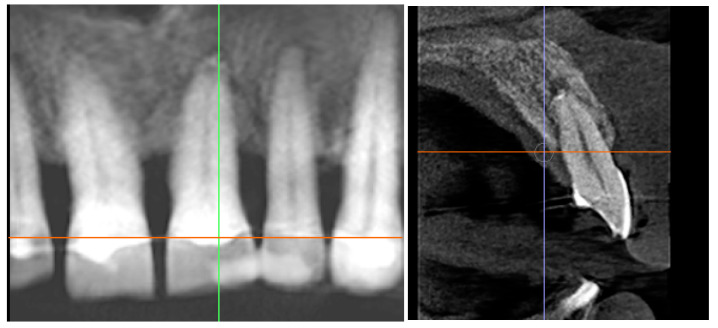
Preoperative CBCT cross-section (baseline).

Cross-sectional CBCT slice depicting vertical bone loss >75% and defect morphology around the compromised root. Orientation markers and a scale bar are included (Figure 14).

**Figure 15:**
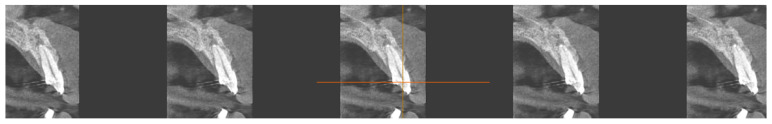
CBCT cross-section at 12 months.

Cross-sectional CBCT slice demonstrating complete bone fill of the previous defect and re-establishment of the periodontal space without evidence of ankylosis. Orientation markers and a scale bar are included (Figure 15).

## 3. Results

### 3.1. Clinical Findings

At baseline, tooth 21 presented with grade III mobility, with horizontal displacement >1 mm and detectable vertical movement. Probing depths ranged from 7 to 9 mm circumferentially, with bleeding on probing, and the gingival tissues showed inflammation consistent with generalized Stage IV, Grade C periodontitis.

Following MIR and completion of orthograde root canal treatment, tooth mobility progressively decreased during the follow-up period. At 6 months, mobility was grade 0 and remained stable at 12 months. At the 12-month evaluation, PPDs were 3-3-3 mm on the buccal and 3-3-4 mm on the palatal surfaces, with no bleeding on probing or suppuration. Gingival contours appeared healthy, with stable marginal levels and no recession in the aesthetic zone.

The tooth was asymptomatic, and the patient reported no discomfort or functional limitation. Occlusion was balanced, and no traumatic contacts on tooth 21 were detected after splint removal. Overall periodontal parameters at baseline and 12 months for tooth #21 and the generalized status. 

### 3.2. Radiographic Findings

Baseline periapical radiographs showed pronounced vertical bone loss around tooth 21, compatible with >75% root length involvement. Early postoperative radiographs (8–12 weeks) revealed an initial increase in trabecular density around the replanted root and a visible PDL-like space. At 24 weeks, progressive mineralization and bone fill were evident, with a more homogeneous trabecular pattern and a stable crestal bone level.

The 12-month periapical radiograph showed radiographic fill of the previous defect, a clear periodontal ligament–like space, and no radiographic signs of ankylosis or external root resorption. The apical region exhibited dense bone surrounding the root apex and a continuous lamina dura.

CBCT cross-sections confirmed the extensive vertical bone defect at baseline and near-complete radiographic fill of the defect at 12 months, with an alveolar crest contour compatible with functional stability. No radiolucent areas suggestive of pathology were detected.

### 3.3. Functional and Aesthetic Outcomes

At 12 months, tooth 21 was in full masticatory function, with normal mobility and no symptoms. The soft tissue profile in the anterior maxilla remained stable, and the aesthetic outcome was satisfactory from the patient’s perspective. No additional surgical or prosthetic interventions beyond the final restoration were required.

## 4. Discussion

This case describes the use of digitally guided MIR to preserve a maxillary anterior tooth with severe periodontal involvement and poor prognosis in a medically complex patient. The 12-month follow-up showed favourable clinical and radiographic outcomes, with resolution of deep pockets, normalization of tooth mobility, and radiographic bone fill around the replanted root.

### 4.1. Case Selection and Prognosis

According to the 2017 AAP/EFP Classification, the patient presented generalized Stage IV, Grade C periodontitis, and tooth 21 exhibited site-specific severe involvement. The prognosis of this tooth was considered poor/unfavourable based on the extent of vertical bone loss, deep pockets, mobility, and the general periodontal condition. Nevertheless, biologically favourable factors—including an intact root, absence of extensive caries or previous endodontic failure, residual palatal bone, and an acceptable crown-to-root ratio—supported an attempt at tooth preservation.

These factors, together with the patient’s systemic condition (heart transplantation, long-term immunosuppression and anticoagulation), made MIR an attractive option compared with extraction and implant placement, which could have required additional bone augmentation and carried a higher surgical burden.

### 4.2. Digital Guidance and Socket Preparation

Digital planning and CAD/CAM-guided socket preparation were central elements in this protocol. The use of CBCT-based planning, a 3D-printed tooth replica, and a tooth-supported surgical guide allowed a controlled osteotomy for a new recipient socket adapted to the root morphology. This approach helped to standardize three-dimensional positioning, reduce operator-dependent variability, and shorten extraoral time by allowing rehearsal with the replica.

These aspects are consistent with previous reports indicating that digital guidance and 3D printing can improve accuracy and reduce extraoral time in autotransplantation and replantation procedures. In periodontally compromised teeth, such precision is particularly relevant because defect morphology and residual bone are often unfavourable.

The biologic drilling protocol without irrigation, using the C3D Cambados guided surgery kit, allowed preservation of autologous bone chips while limiting thermal damage. These chips were then used with xenograft and EMD to support regenerative healing around the replanted root.

### 4.3. Regenerative Approach and Endodontic Protocol

The regenerative strategy combined autologous bone chips, xenograft (Bio-Oss^®^), enamel matrix derivative (Emdogain^®^), and a resorbable collagen membrane (Bio-Gide^®^), following established principles of GTR and EMD-assisted therapy [42,43,44]. This combination aimed to provide space maintenance, scaffold for clot stabilization, and biologic stimulation of periodontal healing.

Endodontic management followed a two-step approach. During the short extraoral phase, apicoectomy and retrograde sealing with Biodentine^®^ were carried out to provide immediate apical closure. After replantation and initial stabilization, a conventional orthograde root canal treatment was performed within the first month. This sequence sought to balance the need to minimize extraoral time with the requirement for thorough canal disinfection, given the recognized association between untreated canals and inflammatory root resorption in replanted teeth. The absence of radiographic signs of resorption at 12 months is consistent with this rationale, although no causal relationship can be inferred from a single case [45,46].

### 4.4. Comparison with Implant-Based Rehabilitation

In many similar clinical scenarios, extraction and implant placement would be considered the standard of care [39,40,41]. In this patient, the combination of severe periodontitis, heart transplantation, and chronic immunosuppression raised concerns about the surgical complexity and potential complications associated with implant placement and possible augmentation procedures.

MIR offered a more conservative, tooth-preserving alternative, maintaining proprioception and the existing soft-tissue architecture [42,47,48]. The purpose of this report is not to propose MIR as a universal alternative to implants, but to illustrate that, in carefully selected cases and with appropriate digital and regenerative support, MIR can provide a viable option that aligns with tooth-preservation principles.

### 4.5. Interpretation of Regenerative Outcomes

The clinical and radiographic findings in this case—reduction in probing depths, normalization of mobility, radiographic bone fill, and presence of a periodontal ligament–like space—are compatible with regenerative healing. However, without histologic examination, it is not possible to confirm true periodontal regeneration. For this reason, the terminology in this manuscript has been deliberately restricted to “radiographic bone fill and clinical improvement compatible with regenerative healing” rather than claiming true regeneration.

## 5. Limitations

This report has several limitations. It describes a single case with a 12-month follow-up, which does not allow conclusions to be drawn about long-term predictability, survival, or late complications such as ankylosis or resorption. Photographic documentation is incomplete; full-mouth intraoral photographs were not systematically obtained, and only one intraoperative photograph was of sufficient quality for inclusion. This limits the visual description of the overall periodontal and occlusal context. Finally, histologic assessment was not performed, so the nature of the attachment cannot be confirmed.

Despite these limitations, the case provides a detailed description of a digitally guided MIR protocol in a tooth with severely compromised periodontal support in a medically complex patient, contributing to the limited literature on this indication.

## 6. Conclusions

In this heart-transplant patient with generalized Stage IV, Grade C periodontitis and a maxillary anterior tooth with poor prognosis, digitally guided modified intentional replantation allowed short-term preservation of the tooth with favourable clinical and radiographic outcomes at 12 months.

The combination of digital planning, controlled socket preparation, atraumatic extraction, extraoral apicoectomy with bioceramic sealing, and guided tissue regeneration supported a stable functional result in this single case. MIR cannot currently be considered a predictable long-term alternative to implants based on existing evidence, but it may be viewed as a tooth-preserving option in carefully selected patients where biological, functional, or systemic factors favour retention of the natural tooth.

Further prospective studies with larger samples and long-term follow-up are required to better define indications, success rates, and comparative outcomes of MIR versus extraction and implant placement.

## Figures and Tables

**Figure 1:**
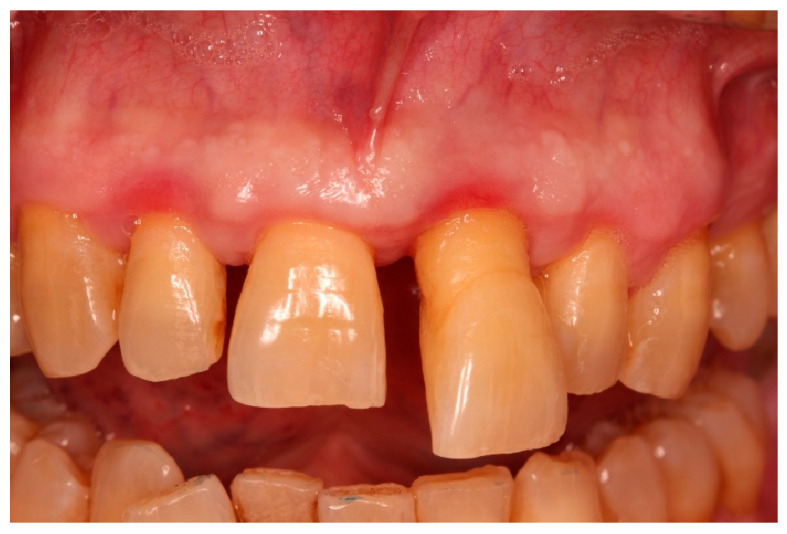
Initial clinical situation.

**Figure 2:**
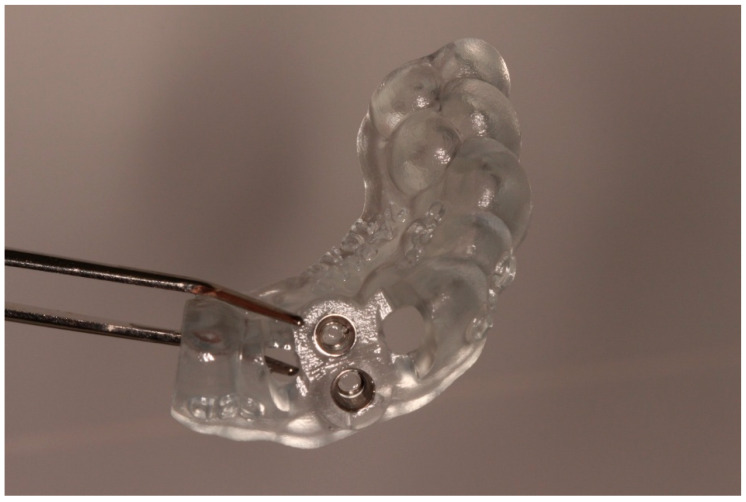
Digitally guided surgical stent for socket preparation.

**Figure 3:**
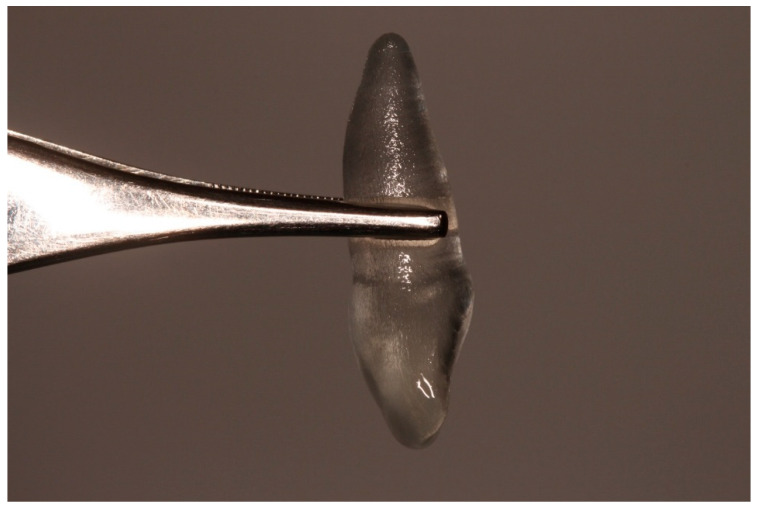
3D-printed replica of the donor tooth.

**Figure 4:**
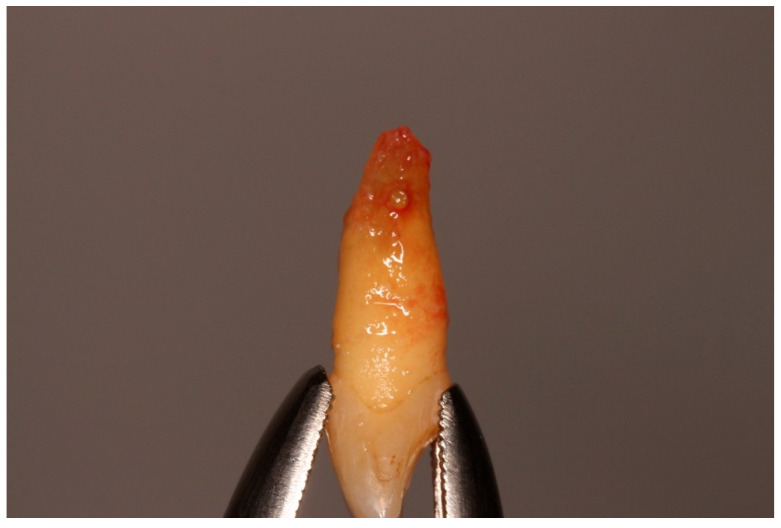
Atraumatic extraction of the compromised tooth.

**Figure 5:**
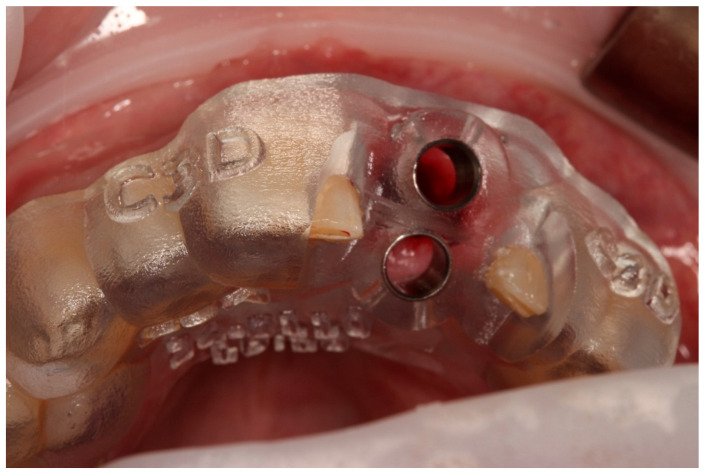
Preparation of the new recipient socket.

**Figure 6:**
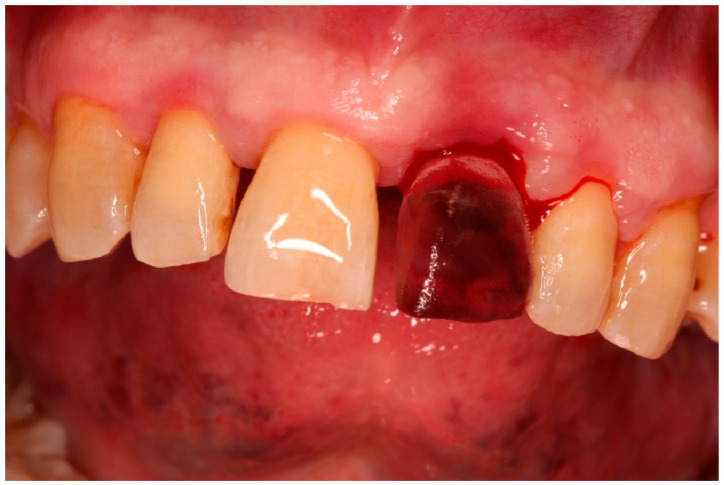
Verification of fit using the 3D tooth replica.

**Figure 7:**
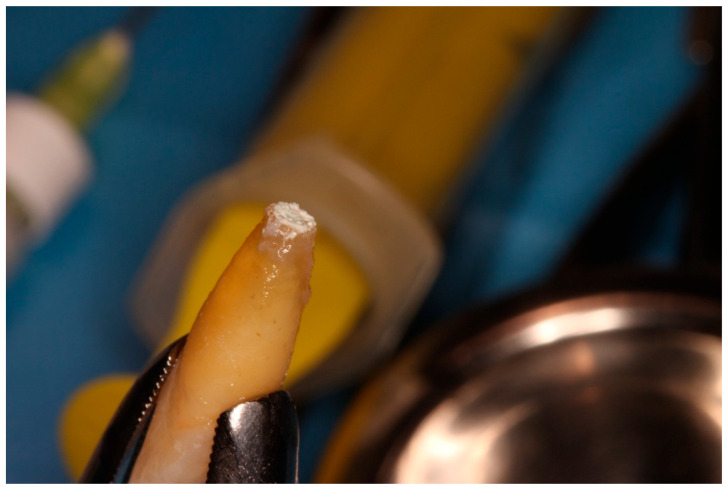
Extraoral apicoectomy and retrograde obturation.

**Figure 8:**
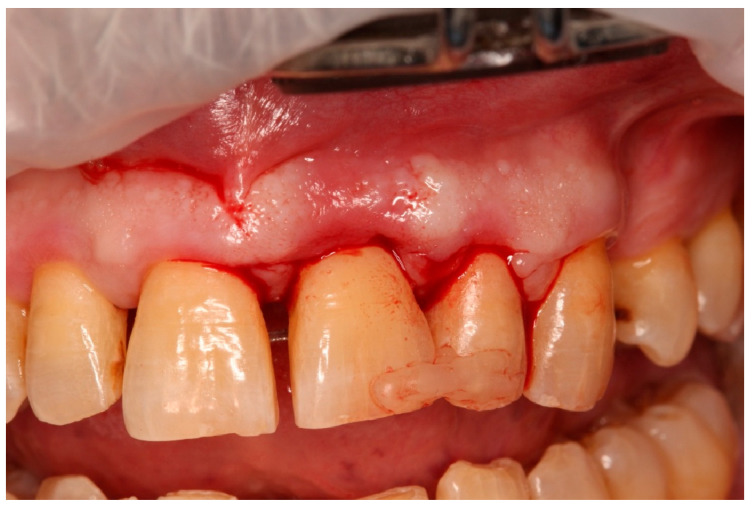
Tooth positioning and semi-rigid splinting in situ.

**Figure 9:**
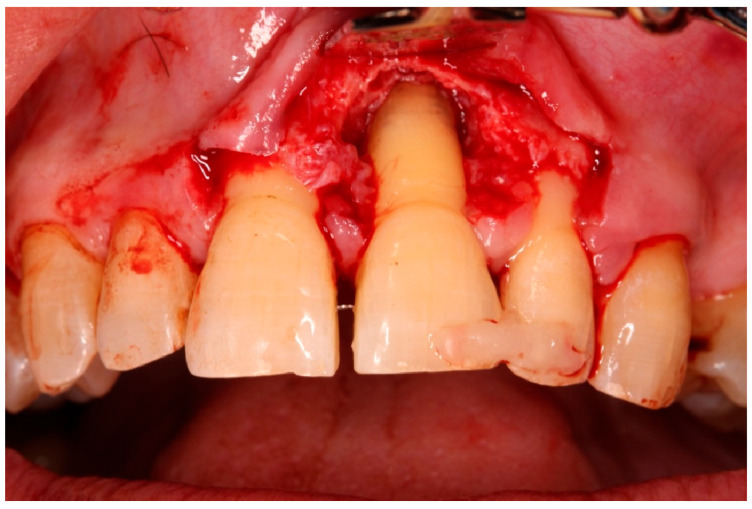
Flap elevation and regenerative phase.

**Figure 10:**
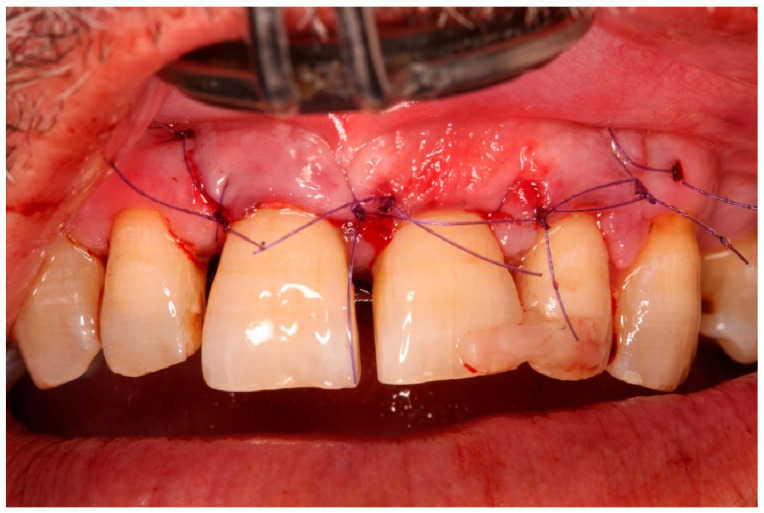
Standardized postoperative periapical radiograph (8–12 weeks).

## Data Availability

Anonymized clinical data underlying this report (periodontal charts, radiographs, and available clinical photographs) are available from the corresponding author upon reasonable request due to confidentiality and ethics.
